# The Baycrest Quick‐Response Caregiver Tool: a Mixed Methods Study in Long‐Term Care

**DOI:** 10.1002/alz70858_102073

**Published:** 2025-12-25

**Authors:** Robert I Madan

**Affiliations:** ^1^ Baycrest, Toronto, ON, Canada; Temerty Faculty of Medicine, Division of Geriatric Psychiatry, University of Toronto, Toronto, ON, Canada

## Abstract

**Background:**

Neuropsychiatric symptoms of dementia (NPS) are common and have negative impacts on health care costs, caregivers, and on quality of life. Behavioural interventions are considered first line for the management of NPS and novel interventions are required. The Baycrest Quick‐Response Caregiver Tool (BQRCT) assists the caregiver to manage NPS in real time. This the tool has been studied in community‐based family caregivers with positive results. The current study examined a new version of the tool for health care professionals in long‐term care (LTC) and assessed its utility and feasibility using mixed‐methodology.

**Method:**

The online training module involved an educational video about NPS, and instructional videos with actors to demonstrate how the tool is used, in addition to an instruction manual and pocket guide. Participants completed the pre‐survey, the training module, and post‐surveys immediately following the training, and after 4 weeks. Survey data included demographics, face‐valid Likert questions for program impact, and feasibility questions.

**Result:**

Participants were recruited from 5 LTC homes. The BQRCT was found to be useful and respondents reported that they would recommend it to other staff. At 4 weeks post intervention, participants reported that their interactions with residents improved as a result of viewing the BQRCT. The qualitative analysis revealed that the tool was informative, practical, and reflected realistic scenarios. Participants found the tool to be easy to understand and use, and it allowed participants to develop empathy through self‐reflection.

**Conclusion:**

The BQRCT was found the be feasible and of utility in the LTC setting. The training module was found to be easy to use and fostered empathy in formal caregivers.

